# Poor Joint Work in the Lower Limbs during a Tennis Forehand Groundstroke after a Cross-Over Step Inhibits an Increase in the Racket Speed

**DOI:** 10.5114/jhk/186535

**Published:** 2024-09-26

**Authors:** Yuta Kawamoto, Takahito Suzuki, Yoichi Iino, Shinsuke Yoshioka, Daisuke Takeshita, Fukashiro Senshi

**Affiliations:** 1 Department of Life Sciences, Graduate School of Arts and Sciences, The University of Tokyo, Tokyo, Japan.; 2 Department of Welfare and Culture, Faculty of Humanities, Okinawa University, Okinawa, Japan.; 3 Japan Women's College of Physical Education, Tokyo, Japan.

**Keywords:** countermovement, inverse dynamics, mechanical energy, run-up, translation and rotation

## Abstract

A forward run-up and stepping are used to accelerate hitting tools or throwing objects in sports. This study aimed to investigate the effect of a forward cross-over step on the speed of a hitting tool by analyzing the joint work and mechanical energy of the whole body and the hitting tool using inverse dynamics. Thirteen advanced tennis players performed forehand groundstrokes at maximum effort with and without a forward cross-over step. From the whole body plus racket perspective, the body-weight-normalized mechanical energy at the start of the hitting motion increased by 1.74 ± 0.42 J·kg^−1^ due to the cross-over step. However, the increase in the magnitude of total negative joint work, primarily attributed to the lower limbs, was 1.38 ± 0.31 J·kg^−1^ due to the cross-over step, conventionally regarded as energy absorption. Consequently, the mechanical energy of the whole body plus the racket at ball impact was comparable between the conditions. Nevertheless, from the segmental perspective, the mechanical work performed by the net shoulder joint force of the playing upper limb with the cross-over step during the hitting motion was greater than that without the cross-over step. Subsequently, the slight increase in the mechanical energy of the playing upper limb plus racket (0.25 ± 0.21 J·kg^−1^) resulted in increased racket speed (4.3%). Considering the comparable total mechanical energy and a resultant increase in racket speed, players and coaches should not overestimate the effect of the forward step on racket speed.

## Introduction

Translational movements of the body, such as a run-up, are used to accelerate hitting tools or throwing objects in sports. For example, in handball throwing the ball velocity is greater with than without a run-up (Wagner et al., 2011, 2012). In javelin throwing, the velocities of the center of mass of elite throwers are higher than those of novice throwers (Bartlett et al., 1996). During bowling in cricket, run-up velocity/speed is positively correlated with ball speed (Ferdinands et al., 2010; Glazier et al., 2000; King et al., 2016; Worthington et al., 2013). Additionally, a previous study indicated that ball velocity increases as run-up velocity increases to the optimal velocity in soccer instep kicking (Andersen and Dörge, 2011). These results indicate that a run-up can enhance the velocity and speed of hitting tools/segments or throwing objects. However, the mechanical cause of the enhancement associated with a run-up and stepping remains unclear.

Considering the whole body plus the hitting tool or the throwing object as a system, one might assume that the run-up would increase the mechanical energy of the entire system. This assumption might include the idea that positive joint work by the lower limbs could be enhanced when the dropping plus landing associated with the forward pre-hop at the end of a run-up is followed by a countermovement, such as the rear knee extension preceded by knee flexion observed during a tennis forehand groundstroke or bowling in cricket (Ferdinands et al., 2014; Iino and Kojima, 2003). Vertical dropping plus landing before jumping increases positive joint work by the lower limbs and jump height when compared with that observed during a countermovement jump that is not preceded by dropping and landing (Moran and Wallace, 2007). However, the effect of a pre-hop landing, where the body drops obliquely, on the body’s mechanical energy can be different from that of a vertical drop and landing due to the involvement of forward motion. For example, the jump height in a vertical countermovement jump preceded by a forward pre-hop is almost the same as that in a normal squat jump (Aeles et al., 2018). This suggests that a forward pre-hop may not significantly increase the mechanical energy of the whole body in the subsequent jump. Moreover, negative knee power production was observed during bowling in cricket (Ferdinands et al., 2014; Middleton et al., 2016) when landing from the delivery jump post run-up (Ferdinands et al., 2010). Therefore, although the run-up can increase the mechanical energy of the whole body plus the hitting tool or the throwing object before the hitting or throwing motion, possible large negative joint work by the lower limbs during the hitting or throwing motion after the run-up could cancel the increase in mechanical energy. To understand the entire scope of the effect of a run-up, it is critical to quantify the joint work and mechanical energy of the whole body plus the hitting tool or the throwing object.

Despite the possible nullification of the increase in total mechanical energy due to a run-up, greater mechanical energy of the playing upper limb (P-UL), with less mechanical energy of the whole body except the P-UL, can lead to more rapid arm swings. It has been qualitatively discussed that the forward momentum gained during a run-up or stepping forward helps accelerate the angular motion of the upper body by thrusting the front lower limb during throwing and bowling (Ferdinands et al., 2010; Matsuo et al., 2001). Although inverse dynamics does not completely reveal the cause of the net joint force (Baltzopoulos, 2021), the change in mechanical energy of the body segment can be quantitatively explained by the mechanical work done at the joints on the segment. The possible increase in kinetic energy of the P-UL could be explained by the increase in mechanical work performed at the shoulder of the P-UL. Mechanical work done by the net shoulder joint force for each direction on the P-UL is determined by the time integration of the product of the net shoulder joint force and the linear velocity of the shoulder joint for each direction. The linear velocity of the trunk kinematically contributes to the linear velocities of the shoulder joint. Therefore, the greater linear velocity of the trunk toward the hitting direction during tennis forehand groundstrokes in the square stance results in greater work performed by the shoulder joint force only in the hitting direction compared with that observed in the open stance (Kawamoto et al., 2019). Tennis players can further move their body in the hitting direction with a square stance using a forward cross-over step (Groppel, 1992), which can also be seen as the last part of the run-up in javelin throwing (Bartlett et al., 1996). In this situation, the further increase in the linear velocity of the trunk toward the hitting direction as a result of the forward cross-over step could increase the work performed by the shoulder joint force compared to that by the square-stance groundstroke without the cross-over step. Thus, quantifying the work done on the P-UL by the shoulder joint could explain the possible increase in mechanical energy of the P-UL.

In this study, we analyzed forehand groundstrokes with and without a forward cross-over step to examine two hypotheses: (1) with a cross-over step, the possible increase in the magnitude of negative joint work due to pre-hop landing may be large enough to cancel the increased mechanical energy of the whole body plus the racket due to the cross-over step; (2) the work performed on the P-UL by the shoulder joint force increases due to the cross-over step; the P-UL plus the racket has more mechanical energy at ball impact with the cross-over step than without it.

## Methods

### Participants

Twelve right-hand-dominant and one left-hand-dominant advanced male tennis players (age: 25.0 ± 2.5 years; body mass: 69.7 ± 9.0 kg; body height: 1.71 ± 0.06 m) participated in the experiment. Participants were recruited through advertisements on social media, and their International Tennis Number (ITF Tennis Development Department, 2004) ranged from two to four, which qualified them as advanced players. All participants provided written informed consent, and the Ethical Review Committee for Experimental Research involving Human Subjects, Graduate School of Arts and Sciences and the College of Arts and Sciences, the University of Tokyo approved all the study procedures (protocol code: No. 450-2; approval date: 27 August 2019).

### Design and Procedures

**Figure 1:**
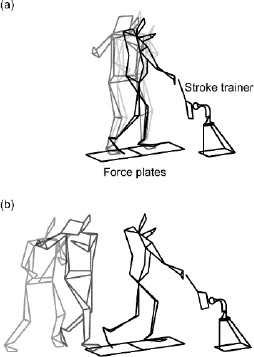
Stick pictures during a groundstroke without the cross-over step (a) and with the cross-over step (b), with force plates and a stroke trainer.

All participants performed forehand groundstrokes under several conditions including a square stance with and without the cross-over step and an open stance without the cross-over step in one day. The data on the square stance with and without the cross-over step were reported in this study, and the data on the square stance without the cross-over step and the open stance without the cross-over step were reported in a previous study (Kawamoto et al., 2019). The order of the square stance with and without the cross-over step was randomly assigned to each participant to ensure a fair comparison. Under the condition without a cross-over step, the feet were set separately on two force plates (Force Plate 9281E, Kistler, Switzerland) that were placed parallel to the hitting direction. Under the condition with a cross-over step, participants stood behind the force plates and pre-hopped onto them using the cross-over step (Figure 1). Participants determined their own start positions so that they were able to hop on the force plates and hit the ball comfortably. The rear foot landing was followed by the front foot landing and the hitting of the ball. A tennis ball was fixed to a stroke trainer (Picotino, Yamakawa, Gunma, Japan) at approximately waist height so that each participant could strike the ball at the same height with and without the cross-over step. Participants held the same racket (GALA, Mizuno, Osaka, Japan) with a semi-western grip and performed four top-spin forehand groundstrokes at maximum effort under each condition, aiming at a target fixed to a pole placed 5 m ahead (Kawamoto et al., 2019). Each participant was allowed to execute forehand groundstrokes with the stroke trainer as a warm-up until acclimating to all conditions. The warm-up lasted for a maximum of 15 min, and forehand groundstrokes were captured for analysis afterward.

### Data Collection and Smoothing

Reflective markers were attached to the relevant anatomical landmarks on each participant, as described in a previous study (Dumas et al., 2007a). Five reflective markers were attached to the racket (Kawamoto et al., 2019). A motion capture system (Motion Analysis Corporation, Santa Rosa, CA, USA) was used to record three-dimensional marker positions at 200 Hz.

To minimize the distortion of the coordinate data due to smoothing through the ball impact (Knudson and Bahamonde, 2001), a quantic spline function was used to smooth the three-dimensional coordinate data of the racket after extrapolating these data (Bahamonde and Knudson, 2003; Chung, 1988) for 15 frames from ball impact (Kawamoto et al., 2019). The time of ball impact was determined by the distance between the markers attached to the racket frame and the stroke trainer. Coordinate data of the body were filtered using a digital fourth-order Butterworth low-pass filter with a zero phase lag. The cut-off frequencies were determined via residual analysis (Winter, 2009). Force data recorded at 2,000 Hz were downsampled and filtered using a digital fourth-order Butterworth low-pass filter with zero phase lag. Since filtering the kinetic data with a higher cut-off frequency than that for the kinematic data exaggerates the fluctuations in the lower joint torques shortly after contact (Bezodis et al., 2013; Kristianslund et al., 2012), the cut-off frequency for the force data was determined by averaging the cut-off frequencies for lower-limb markers.

### Coordinate Systems and Calculation of Work

The whole body and the racket were modeled as 16 rigid-link segments: a head, a torso, a pelvis, upper arms, forearms, hands, thighs, legs, feet, and a racket. Body segment coordinate systems were constructed, and body segment inertia parameters were estimated, as previously described (Dumas et al., 2007a, 2007b). The orthogonal joint coordinate systems of the upper limb and inertia parameters of the racket were determined according to a previous study (Kawamoto et al., 2019). The torques of the lower limb joints were anatomically expressed in the coordinate systems of the distal segments (Akutagawa and Kojima, 2005; Chappell et al., 2002; Davis et al., 1991), and the shoulder joint force was expressed in the global coordinate system. The Y-axis was directed toward the hitting direction, the Z-axis was directed vertically upward, and the X-axis was directed toward the right with the player facing the hitting direction. Joint work was determined based on the time integration of the joint torque power, which is the dot product of the torque and joint angular velocity at each joint. The work done on the P-UL plus the racket by the shoulder joint force was determined based on the time integration of the joint force power, which is the product of the joint force and velocity of the shoulder joint for each direction. The work done on the P-UL plus the racket by the shoulder joint torque was determined by the time integration of the segment torque power, which is the dot product of the joint torque and angular velocity of the upper arm. All numerical integrations to determine work were conducted from the start of the hitting motion until ball impact.

### Hitting Motion

To compare the two conditions as fairly as possible, we sought common action events to define the beginning and the end of the hitting motion. With the cross-over step, the center of mass of the whole body plus the racket continued descending after rear foot landing from a pre-hop and subsequently rose before ball impact. Without the cross-over step, the center of mass also showed a similar movement pattern during the hitting motion. Under both conditions, rear knee flexion with extension torque accompanied the descending movement of the center of mass, leading to negative joint torque power at the rear knee. Thus, rear foot landing was chosen as the beginning of the hitting motion with the cross-over step, while the onset of the falling of the center of mass of the whole body was chosen as the beginning of the hitting motion without the cross-over step. Under both conditions, the ball impact was chosen as the end of the hitting motion.

### Statistical Analysis

Kinetic and mechanical energy as well as mechanical work were normalized with body weight. Kinematic and kinetic data of each participant were determined by averaging the data of four trials, and the mean data of each condition was calculated by averaging the determined kinematic and kinetic data of all participants. Statistical analyses were performed using Excel for Microsoft 365 (ver. 2211, Microsoft Corporation, Redmond, WA, USA). A two-tailed paired t-test was used to compare the 16 variables obtained with and without the cross-over step. Adjusted p-values and 95% confidence intervals of the mean difference were calculated based on a previous study (Serlin, 1993) using the Holm-Bonferroni method with an overall type I error rate of 0.05.

## Results

The racket head center speed at ball impact was greater with the cross-over step than without the cross-over step (adjusted *p* = 0.030; Table 1). The racket head center speed was, on average, 4.3% higher with the cross-over step than without the cross-over step. Other kinematic variables of the racket and the trunk or the duration of the hitting motion are listed in the supplemental file (Supplemental Table 1).

**Table 1:** Racket speed with mechanical energy and work normalized by body weight (mean ± *SD*, with *p*-value and 95% CI adjusted using the Holm-Bonferroni method and Cohen’s *d*).

	With cross-over	Without cross-over	*p*	*d*	95% CI
Speed of the racket head center at ball impact [m·s^−1^]	30.3 ± 2.67*	29.1 ± 2.72	0.030	0.450	[0.10, 2.33]
Mechanical energy [J·kg^−1^]					
Whole-body plus the racket					
at the start of hitting motion	11.3 ± 0.58*	9.57 ± 0.42	< 0.001	3.430	[1.32, 2.16]
at ball impact	11.4 ± 0.52	11.2 ± 0.37	0.545	0.423	[−0.15, 0.53]
P-UL plus the racket					
at the start of hitting motion	0.74 ± 0.06*	0.68 ± 0.05	< 0.001	1.106	[0.03, 0.10]
at ball impact	2.54 ± 0.42*	2.28 ± 0.43	0.008	0.598	[0.06, 0.45]
Racket					
at the start of hitting motion	0.08 ± 0.01	0.08 ± 0.01	1	0.179	[−0.00, 0.01]
at ball impact	1.35 ± 0.30*	1.23 ± 0.30	0.011	0.396	[0.02, 0.22]
Work done on the P-UL plus the racket [J·kg^−1^]					
Shoulder joint force					
total	0.64 ± 0.29*	0.47 ± 0.32	0.022	0.551	[0.02, 0.32]
(hitting direction)	0.53 ± 0.15*	0.35 ± 0.16	0.002	1.166	[0.06, 0.30]
Shoulder joint torque					
total	1.26 ± 0.25	1.26 ± 0.29	1	−0.008	[−0.12, 0.12]
Joint work [J·kg^−1^]					
Sum of lower limb joints					
positive	2.18 ± 0.47	1.93 ± 0.38	0.400	0.588	[−0.15, 0.66]
negative	−2.33 ± 0.45*	−0.91 ± 0.35	< 0.001	−3.516	[−1.73, −1.10]
net	−0.15 ± 0.55*	1.02 ± 0.29	< 0.001	−2.659	[−1.64, −0.69]
Total of all joints					
positive	3.51 ± 0.80	3.16 ± 0.75	0.126	0.459	[−0.07, 0.78]
negative	−2.86 ± 0.60*	−1.49 ± 0.48	< 0.001	−2.539	[−1.69, −1.06]
net	0.65 ± 0.71*	1.67 ± 0.53	< 0.001	−1.633	[−1.51, −0.53]

* statistically significant; SD: standard deviation; CI: confidence interval; P-UL: playing upper limb

**Figure 2:**
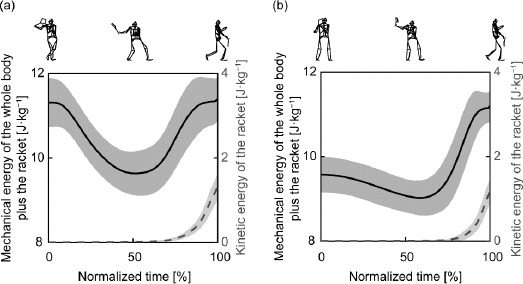
The ensemble average of mechanical energy of the whole body plus the racket (solid black line) and kinetic energy of the racket (dashed grey line) normalized by body weight with the cross-over step (a) and without the cross-over step (b). Rear lower limb landing occurs at 0% with the cross-over step, and the center of mass of the whole body plus the racket begins to fall at 0% without the cross-over step. Ball impact occurs at 100% under both conditions.

The mechanical energy of the whole body plus the racket was higher at the start of the hitting motion with the cross-over step than without the cross-over step (adjusted *p* < 0.001; Table 1), but there was no significant difference between the mechanical energy of the whole body plus the racket at ball impact with and without the cross-over step (Table 1 and Figure 2).

The difference between the mechanical energy of the whole body plus the racket at the start of the hitting motion with and without the cross-over step (1.74 ± 0.42 J·kg^−1^) was, on average, approximately 150% of the kinetic energy of the racket at ball impact without the cross-over step (Figure 2).

At the start of the hitting motion, the mechanical energy of the P-UL plus the racket was greater with the cross-over step than without the cross-over step (adjusted *p* < 0.001; Table 1). The mechanical energy of the racket, as well as the mechanical energy of the P-UL plus the racket, was greater with the cross-over step than without the cross-over step at ball impact (racket: adjusted *p* = 0.011; P-UL plus racket: adjusted *p* = 0.008). The work done on the P-UL by the shoulder joint force in the hitting direction and the total work done by the shoulder joint forces were greater under the cross-over step than the non-cross-over step condition (hitting direction: adjusted *p* = 0.002; total: adjusted *p* = 0.022). No significant difference in the total work done on the P-UL by the shoulder joint torque was observed between the two conditions.

**Figure 3:**
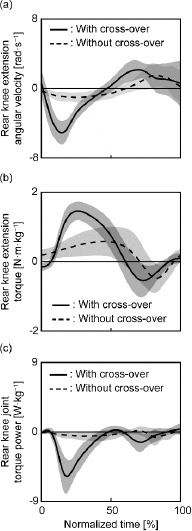
The ensemble average of extension angular velocity (a), extension torque (b), and joint torque power about the extension/flexion axis (c) at the rear knee. The torque and power were normalized by body weight. The solid line shows the condition with the cross-over step, and the dashed line shows the condition without the cross-over step.

With and without the cross-over step, positive joint work was greatest at the rear hip, followed by the lumbosacral joint (Supplemental Table 2). There was no significant difference in the positive joint work by the lower limbs or the total positive joint work at all joints between the two conditions (Table 1). However, the amount of negative joint work done by the lower limbs and total negative joint work done at all joints was greater with the cross-over step than that without the cross-over step (adjusted *p* < 0.001; Table 1). As a result, the total net joint work of all joints as well as net joint work by the lower limbs was lower with the cross-over step than without it (adjusted *p* < 0.001). With the cross-over step, the magnitude of negative joint work was largest at the rear knee compared with that of other joints. At the rear knee, flexion and the exertion of extension torque were followed by extension and the exertion of flexion torque with and without the cross-over step (Figure 3). Therefore, the knee joint torque power was negative throughout most parts of the hitting motion under both the cross-over step and without the cross-over step conditions.

## Discussion

**Figure 4:**
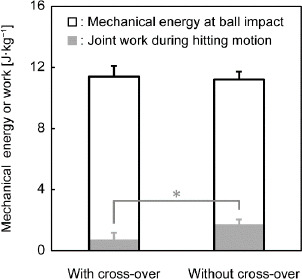
The mechanical energy of the whole body plus the racket at ball impact and total net joint work during hitting motion normalized by body weight with and without the cross-over step. * adjusted p < 0.05, indicating a significant difference

Despite the greater mechanical energy of the whole body plus the racket at the start of the hitting motion under the cross-over step condition, the cross-over step was associated with smaller total net joint work of all joints due to considerable total negative joint work, resulting in similar mechanical energy of the whole body plus the racket at ball impact (Figure 4).

However, the work done on the P-UL by the shoulder joint force was greater with the cross-over step than without the cross-over step, leading to an increase in the mechanical energy of the P-UL plus the racket. Consequently, the cross-over step increased the racket head center speed at ball impact by 4.3% when compared to the no cross-over step condition.

### Enhancement of Racket Speed

At ball impact, the cross-over step increased the speed of the racket head center by an average of 4.3%. Using a fixed ball and hitting from a stationary position in this study could exclude irrelevant kinematic variability in response to the incoming ball and make it easier to identify performance enhancement due to the cross-over step.

### Energetics of the Whole Body plus the Racket

The numerical difference between the mechanical energy of the whole body plus the racket at the start of the hitting motion with and without the cross-over step was approximately 150% of the kinetic energy of the racket at ball impact without the cross-over step (Figure 2). However, the mechanical energy of the whole body with the cross-over step drastically decreased during the first half of the hitting motion. The cross-over step increased the magnitude of negative joint work by the lower limbs without increasing the positive joint work by the lower limbs, resulting in a decrease in net joint work by the lower limbs (Table 1). The magnitude of the negative joint work was largest at the rear knee with the cross-over step compared to that of the other joints. At the rear knee, flexion torque was exerted after the exertion of extension torque without the cross-over step, consistent with the findings of previous studies (Iino and Kojima, 2001, 2003). Similarly, we observed such flexion torque with the cross-over step. Although, with or without the cross-over step, the knee flexed and subsequently extended like in a countermovement jump (Moran and Wallace, 2007), a nearly simultaneous transition from extension torque to flexion torque resulted in negative power exertion through most parts of the hitting motion. Furthermore, the magnitude of the negative power was substantial during the hitting motion following the cross-over step (Figure 3), contributing to the greater magnitude of negative joint work by the lower limbs than observed without the cross-over step. The increase in the amount of negative work by the lower limbs due to the cross-over step (1.42 ± 0.31 J·kg^−1^) was considerable enough to account for the increase in the amount of total negative work at all joints (1.38 ± 0.31 J·kg^−1^). As a result, the reduction in total net joint work caused by the cross-over step can be attributed to negative joint work at the lower limbs.

Due to the smaller total net joint work, despite the greater mechanical energy of the whole body plus the racket at the start of the hitting motion under the cross-over step condition than that under the non-cross-over step condition, the mechanical energy of the whole body plus the racket at ball impact was similar between the two conditions (Figure 4).

### Energetics of the P-UL plus the Racket

**Supplemental Figure 1:**
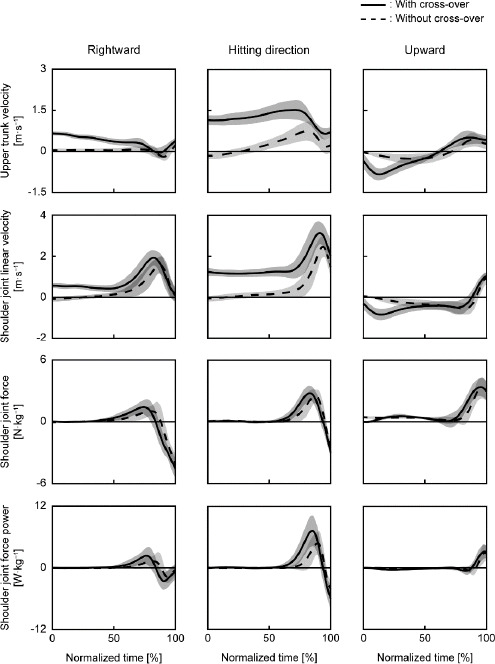
The ensemble average of the velocity of the center of mass of the torso, linear velocity of the shoulder joint, net joint force at the shoulder normalized by body weight, and joint force power at the shoulder normalized by body weight. The solid line shows the condition with the cross-over step, and the dashed line shows the condition without the cross-over step.

The cross-over step increased the mechanical energy of the P-UL plus the racket at ball impact without increasing the mechanical energy of the entire body. An increase in mechanical energy of the P-UL plus the racket leads to an increase in the speed of the racket at ball impact owing to the cross-over step. The effect of the cross-over step on the mechanical energy of the P-UL plus the racket can be considered an extension of the relationship between the square-stance stroke and the open-stance stroke. In the square stance, the linear velocity of the torso increases due to the step forward in the hitting direction, and the work done by the shoulder joint force in the hitting direction is greater than that in the open stance, although the amount of work done by the shoulder joint torque is similar (Kawamoto et al., 2019). In this study, the increased linear velocity of the torso in the hitting direction due to the cross-over step, which kinematically contributed to the linear velocity of the shoulder, seemed to increase the joint force power and work done by the shoulder joint force (Supplemental Figure 1). Since the work done on the P-UL by the shoulder joint torque (Table 1) and joint work by the other joints of the P-UL plus the racket (Supplemental Table 2) were similar between the cross-over step and without cross-over step conditions, greater work done by the shoulder joint force resulted in greater mechanical energy of the P-UL plus the racket with the cross-over step than without the cross-over step. This resulted in faster movement of the P-UL plus the racket with the cross-over step compared to the non-cross-over step condition. However, even if the increased work done by the shoulder joint force in the hitting direction due to the cross-over step had been completely converted and added to the kinetic energy of the racket at ball impact without the cross-over step (Table 1 and Supplemental Table 1), the racket speed could have increased by an average of 7.6%. Therefore, although the work done by the shoulder joint force and the resultant speed of the racket increased due to the cross-over step, these increases were small compared to the increase in the mechanical energy of the whole body plus the racket at the start of the hitting motion.

### Optimal Run-Up Speed

Although the initial mechanical energy of the whole body plus the racket increased, the net joint work during the hitting motion decreased due to the cross-over step. The balance between the initial mechanical energy and the net joint work during the hitting motion could change with the run-up speed. The best balance would lead to the highest mechanical energy of the whole body, including the playing limb at ball impact. Therefore, the existence of optimal run-up speed, which has been described in a previous study on soccer kicking (Andersen and Dörge, 2011), can be partly attributed to the aforementioned balance.

### Strengths and Limitations of the Study

To our knowledge, this is the first study to investigate the effect of a cross-over step on the speed of a hitting tool using the application of the work-energy theorem. Due to the time constraints and the limited number of steps available during a tennis groundstroke rally, we focused exclusively on the cross-over step, which can be assumed as a one-step run-up. Conversely, in other sports movements, such as soccer free kicking or javelin throwing, the player can use more time and steps for the run-up. Therefore, future studies investigating a wide range of run-up speeds are required to understand the effect of a run-up on hitting tools or throwing objects comprehensively.

## Conclusions

This study aimed to investigate the effect of a cross-over step on the speed of a hitting tool. At the start of the hitting motion, compared with the condition without the cross-over step, the mechanical energy of the P-UL plus the racket was slightly greater, and that of the whole body plus the racket was considerably greater with the cross-over step. The work done on the P-UL by the net shoulder joint force increased during the hitting motion, while the magnitude of negative joint work at the lower limbs considerably increased due to the cross-over step. Although the increased negative work almost canceled out the increased mechanical energy of the whole body plus racket due to the cross-over step at the start of the hitting motion, the work done on the P-UL plus racket increased the mechanical energy of the P-UL plus the racket and thus racket speed (4.3%).

## Figures and Tables

**Supplemental Table 1:** Kinematic and temporal data and kinetic energy normalized by body weight (mean ± *SD*).

	With cross-over	Without cross-over
Velocity of the racket head center in the hitting direction at ball impact [m·s^−1^]	27.5 ± 2.1	26.2 ± 1.7
Velocity of the center of mass of the torso in the hitting direction [m·s^−1^]		
at the start of hitting motion	1.15 ± 0.18	−0.16 ± 0.11
at ball impact	0.70 ± 0.19	0.24 ± 0.26
Velocity of the center of mass of the torso in the upward direction [m·s^−1^]		
at the start of hitting motion	−0.36 ± 0.27	−0.04 ± 0.03
at ball impact	0.42 ± 0.16	0.27 ± 0.20
Peak angular velocity around its superior/inferior axis [rad·s^−1^]		
about the torso	12.7 ± 1.7	12.1 ± 1.6
about the pelvis	8.64 ± 1.36	7.91 ± 1.33
Duration from the start of hitting motion to ball impact [s]	0.57 ± 0.10	0.80 ± 0.17
Kinetic energy [J·kg^−1^]		
P-UL plus the racket		
at the start of hitting motion	0.05 ± 0.02	0.03 ± 0.01
at ball impact	2.04 ± 0.43	1.78 ± 0.44
Racket		
at the start of hitting motion	0.004 ± 0.002	0.006 ± 0.003
at ball impact	1.32 ± 0.30	1.20 ± 0.30

SD: standard deviation; P-UL: playing upper limb

**Supplemental Table 2:** Joint work normalized by body weight (mean ± *SD* J·kg^−1^).

	With cross-over		Without cross-over	
	positive	negative	positive	negative
P-UL shoulder	0.27 ± 0.10	−0.08 ± 0.04	0.31 ± 0.10	−0.07 ± 0.03
P-UL elbow	0.03 ± 0.03	−0.09 ± 0.05	0.03 ± 0.03	−0.09 ± 0.05
P-UL wrist	0.03 ± 0.01	−0.05 ± 0.03	0.02 ± 0.01	−0.04 ± 0.03
Joint between the racket and the hand	0.01 ± 0.00	−0.04 ± 0.02	0.01 ± 0.00	−0.04 ± 0.03
NP-UL shoulder	0.33 ± 0.19	−0.01 ± 0.01	0.32 ± 0.19	−0.01 ± 0.01
NP-UL elbow	0.08 ± 0.05	−0.01 ± 0.01	0.07 ± 0.05	−0.01 ± 0.01
NP-UL wrist	0.002 ± 0.002	−0.002 ± 0.001	0.002 ± 0.002	−0.001 ± 0.001
Front hip	0.36 ± 0.09	−0.30 ± 0.11	0.29 ± 0.13	−0.14 ± 0.08
Front knee	0.16 ± 0.13	−0.37 ± 0.10	0.21 ± 0.15	−0.08 ± 0.07
Front ankle	0.07 ± 0.08	−0.13 ± 0.05	0.08 ± 0.06	−0.03 ± 0.02
Rear hip	1.27 ± 0.30	−0.19 ± 0.15	1.11 ± 0.27	−0.20 ± 0.14
Rear knee	0.14 ± 0.07	−0.88 ± 0.18	0.09 ± 0.07	−0.33 ± 0.20
Rear ankle	0.19 ± 0.13	−0.46 ± 0.19	0.15 ± 0.10	−0.12 ± 0.07
Neck	0.02 ± 0.01	−0.02 ± 0.01	0.02 ± 0.01	−0.01 ± 0.00
Lumbosacral	0.57 ± 0.18	−0.25 ± 0.18	0.45 ± 0.21	−0.31 ± 0.17

SD: standard deviation; P-UL: playing upper limb; NP-UL: non-playing upper limb
